# Hydroxyethoxy phenyl butanone, a new cosmetic preservative, does not cause bacterial cross-resistance to antimicrobials

**DOI:** 10.1099/jmm.0.001147

**Published:** 2020-03-18

**Authors:** Rebecca Wesgate, Florence Menard-Szczebara, Ahmad Khodr, Sylvie Cupferman, Jean-Yves Maillard

**Affiliations:** ^1^​ Cardiff School of Pharmacy and Pharmaceutical Sciences, Cardiff University, Cardiff, UK; ^2^​ L’Oréal Research and Innovation, Chevilly-Larue, France

**Keywords:** resistance, antibiotics, biocides, predictive protocol

## Abstract

**Introduction.:**

Biocide-induced cross-resistance to antimicrobials in bacteria has been described and is a concern for regulators. We have recently reported on a new protocol to predict the propensity of biocide to induce phenotypic resistance in bacteria.

**Aim.:**

To measure bacterial propensity to develop antimicrobial resistance following exposure to a new cosmetic preservative developed by L’Oréal R and I.

**Methodology.:**

Well-established antimicrobials including triclosan (TRI) and benzalkonium chloride (BZC) and a new molecule hydroxyethoxy phenyl butanone (HEPB) were investigated for their antimicrobial efficacy, effect on bacterial growth, and their potential to induce resistance to chemotherapeutic antibiotics using a new predictive protocol.

**Results.:**

The use of this predictive protocol with *Staphylococcus aureus *, *Escherichia coli* and *Pseudomonas aeruginosa* showed that TRI and BZC significantly affected bacterial growth, MICs and minimum bactericidal concentrations (MBCs). There was no change in antibiotic susceptibility profile following exposure to BZC, but *E. coli* became intermediate resistant to tobramycin following treatment with TRI (0.00002 % w/v). HEPB did not change the antimicrobial susceptibility profile in *P. aeruginosa* and *S. aureus* but *E. coli* became susceptible to gentamicin. TRI exposure resulted in bacterial susceptibility profile alteration consistent with the literature and confirmed the use of TRI as a positive control in such a test.

**Conclusion.:**

Data produced on the propensity of a molecule to induce bacterial resistance is useful and appropriate when launching a new preservative.

## Introduction

Biocides are antimicrobials that are used for antisepsis, preservation and disinfection. They play an important role in infection control regimens for eliminating pathogenic micro-organisms on hands and inanimate surfaces [1]. Their role for preservation is equally important and their use is widespread in different industrial environments (e.g. food, pharmaceuticals, materials, etc.). Despite the control of existing and novel biocide substances commercialized in the European market, there has been an increased usage of biocides, particularly in consumer-based and healthcare products [2, 3]. The overuse and/or sometimes misuse of biocides have been seen as a possibility to exacerbate emerging antimicrobial resistance in bacteria [2, 3]. The number of reports on emerging multidrug-resistant bacteria isolated from food and water environments is increasing [4, 5]. The possibility of biocide-induced antimicrobial resistance is of particular concern [2, 3, 6–8]. The European Biocidal Product Regulation (effective from 1 September 2013) request manufacturers to provide information on the impact of their product and antimicrobial resistance (articles 19-b) ii, 37 and 47-1/b/. With this in mind, it has become necessary to test not only biocide efficacy against targeted micro-organisms but also for the risk associated with biocide/biocidal product usage on emerging resistance in bacteria [9, 10]. We have recently reported the use of protocol based on exposure to ‘during use’ concentration of biocides to inform the ability of bacteria to develop resistance and cross-resistance to antimicrobials. This method is based on evaluating the changes in MIC, minimum bactericidal concentration (MBC) and antibiotic clinical susceptibility profile of target bacteria before and after exposure to a biocide/biocidal product, with the stability of phenotypic changes, if any, evaluated. The ‘during use’ exposure refers to the product concentration and contact time during usage [9]. The use of this protocol enabled us to show how targeted bacteria responded to the effect of cationic biocides such as benzalkonium chloride (BZC) and chlorhexidine preserved products (e.g. shampoo, eye liner) [10], and formulated and unformulated hydrogen peroxide [10]. In these studies, TRI was used as a ‘positive’ control in that it produced significant changes in the susceptibility phenotype of targeted Gram-positive and Gram-negative bacteria. BZC is a quaternary ammonium compound used as a preservative in formulations although it possesses wetting and solubilizing properties [11]. TRI is a chlorophenol that has been used in a wide variety of applications, spanning from preservatives in household products like vacuum cleaners to disinfectants, make-up and industrial cleaning agents and cosmetics [12–14].

To date, no new antimicrobial molecules have been tested with this protocol to investigate possible development of bacterial resistance. The present study reports on the use of this methodology to test the bacterial response to a novel molecule hydroxyethoxy phenyl butanone (HEPB) to be used as a preservative agent.

## Methods

### Micro-organisms and growth conditions

*Pseudomonas aeruginosa* ATCC 19429 was cultured from working stocks to 10 ml tryptone soya broth (TSB; Oxoid, Basingstoke, UK) and incubated for 24 h at 30 °C (±1 °C). *Staphylococcus aureus* NCIMB 9518 and *Escherichia coli* ATCC 8739 were cultured from working stocks to 10 ml TSB and incubated for 24 h at 35 °C (±1 °C). Broth cultures were centrifuged at 2500 ***g*** for 15 min and pellets re-suspended in tryptone sodium chloride (TSC; 1 g tryptone l^−1^ and 8.5 g sodium chloride l^−1^) adjusted to ~10^8^ c.f.u. ml^−1^.

## Antimicrobials

A preservative molecule obtained from L’Oréal R and I, hydroxyethoxy phenyl butanone – 020 001 (HEPB), triclosan (TRI; Medex, Warwickshire, UK) and benzalkonium chloride (BZC, alkyl distribution C8H17 to C16H33; Fisher Scientific, Leicestershire, UK) were investigated. The highest HEPB tested concentration of 2 % w/v almost corresponded to three times the prospective in use concentration for this new preservative in formulae. To solubilize HEPB and TRI, 5 % v/v dimethyl sulfoxide (DMSO; Fisher Scientific, Leicestershire, UK) was used. The lack of toxicity of the solubilizing agent was evaluated using the Bioscreen C analyser (Oy Growth Curves AB, Helsinki, Finland). The use of 5 % v/v DMSO had no detrimental effect on the growth of the test strains (Fig. S1, available in the online version of this article). The activity of all preservatives was quenched with a neutralizing solution consisting of 1.5 % v/v Tween 80 and 3 % w/v lecithin (Fisher scientific, Leicestershire, UK). Neutralizer efficacy and toxicity were tested following the experimental conditions of Knapp *et al*. [15]. Briefly, 1 ml of a standardized bacterial inoculum (1×10^8^ c.f.u. ml^−1^) was added to 9 ml of neutralizer for or deionized water (control) for 5 min. Survivors were then serially diluted in TSC, and enumerated using the standardized Miles and Misra method [16]. The neutralizer was considered not toxic since <1 log_10_ reduction was observed following bacterial exposure to the neutralizer. The efficacy of the neutralizer to quench the activity of the biocides was evaluated by adding 1 ml of bacteria (1×10^8^ c.f.u. ml^−1^) to a suspension composed of 1 ml of the highest concentration of biocide tested and 8 ml of neutralizer. After a 5 min contact time, surviving bacteria were serially diluted in TSC and enumerated as described above. As a control, the 8 ml of neutralizer was replaced with 8 ml of deionized water. The neutralizer used was efficacious against all biocides as <1 log_10_ reduction was observed in the neutralized biocide suspension.

The following antibiotics were used: ampicillin (10 µg), ticarcillin/clavulanic acid (75/10 µg), ciprofloxacin (1 µg), tobramycin (10 µg), ceftazidime (30 µg), cefotaxime (30 µg), tetracycline (30 µg) and gentamicin (10 µg). All antibiotics were purchased from Oxoid.

## Growth kinetics

Bacterial growth kinetics with or without preservative was determined using the Bioscreen C microbial growth analyser based on Gomez Escalada *et al*. [17]. Each well of the Bioscreen plate contained a total volume of 400 µl of bacterial suspension (~2×10^6^ c.f.u. well^−1^) in TSB. The Bioscreen was run for 24 h at 25 °C (±1 °C) and readings were taken using a wideband filter (420–580 nm) every 15 min preceded by 10 s shaking. Controls consisted of TSB, bacteria in TSB±5 % v/v DMSO.

### Antimicrobial activity and baseline data

A novel predictive protocol was used to evaluate the propensity of micro-organisms to develop resistance to antimicrobials [2]. The protocol is based on the evaluation of the antimicrobial susceptibility profile before and after exposure to an antimicrobial mimicking ‘during use’ conditions of the antimicrobial and establishing the stability profile of any altered antimicrobial susceptibility profile.

MICs were determined before and after biocide exposure with the British Standard EN ISO: 20776–1 (2006) [18] microdilution protocol. The test inoculum consisted of washed cells (i.e. stationary phase) with a bacterial concentration ~10^6^ c.f.u., inoculated in TSB in decreasing concentrations of the biocide in 96-well plates (ThermoFisher Scientific, Leicestershire, UK). The MIC was taken as the lowest concentration of the preservative that showed no growth after 24 h incubation at 37 °C (±1 °C) or 25 °C (±1 °C).

MBCs were determined by plating out 20 µl of test suspension from well of the MIC 96-well plate where no bacterial growth was observed and the two lowest biocide concentrations at which growth was observed on media (TSA) containing 10 % v/v neutralizer. After 24 h incubation at 37 °C (±1 °C), the MBC was defined as the lowest preservative concentration where no bacterial growth occurred.

### Antibiotic susceptibility testing

Antibiotic susceptibility testing was performed according to the disk diffusion assay described by the British Society for Antimicrobial Chemotherapy (BSAC). The clinical interpretation of the zone of inhibition was based on the work of Andrews [19].

### Exposure to antimicrobial compounds

Micro-organisms were exposed to preservatives in a suspension test based on the British Standard EN 1276 (2009)[20]. Briefly, 1 ml of an overnight washed bacterial (1×10^8^ c.f.u. ml^−1^) suspension in TSC was added to 9 ml of the of a preservative/product (diluted in diH_2_0) for 24 h at 20 °C (±1 °C). The concentrations chosen were within the concentrations affecting growth or 1/10 of the MIC: for *P. aeruginosa *: 0.00063 % w/v BZC, 0.0001 % w/v TRI; for *S. aureus* 0.00008 % w/v BZC, 0.00005 % w/v TRI; and for *E. coli* 0.00016 % w/v BZC, 0.00002 % w/v TRI. For HEPB an arbitrary 0.2 % w/v corresponding to 1/10 of the highest concentration tested was used for all strains since no MIC could be found for this compound. Following exposure, the test micro-organisms were filtered through a 0.2 µm filter and washed with 5 ml neutralizer followed by 5 ml TSC. The filter was then placed in a bottle with 5 ml TSC and 5 g glass beads and vortexed for 1 min to recover survivors. Susceptibility testing was performed on all survivors after exposure and results were compared to baseline data.

### Reproducibility

Tests were conducted in triplicate on three separate occasions with exception of the microbial growth analyses, that were conducted twice. No statistical analysis was conducted on antibiotic breakpoints as only the clinical resistance breakpoint given by BSAC [19] was of major interest.

## Results

### Growth kinetics

The effect of the antimicrobial compounds on the growth of the test microorganisms is presented as Supplementary Material (Fig. S2–S10). Changes in growth pattern were informed by increasing lag phase, change in observed growth rate during the exponential phase, and final OD values. The control containing 5 % v/v DMSO did not affect the growth of test micro-organisms. *E. coli* and *S. aureus* started their exponential phase faster than *P. aeruginosa *. Different concentrations of BZC inhibited the growth of the test micro-organisms: ≥0.013 % w/v for *P. aeruginosa *, ≥0.0031 % w/v for *E. coli* and ≥0.00078 %w/v for *S. aureus* (Figs S﻿2, S5, S8). Overall, *S. aureus* growth was severely affected by the QAC concentrations tested (Fig. S8). BZC also affected the growth of *P. aeruginosa* at concentrations between 0.0063 and 0.00039 % w/v, *E. coli* at concentrations between 0.0016 and 0.00078 % w/v and *S. aureus* at a concentration of 0.00039 % w/v.

TRI affected but did not inhibit the growth of *P. aeruginosa* (Fig. S3). The effect of TRI was more pronounced against *E. coli* with all concentrations tested affecting bacterial growth (Fig. S6). TRI affected the growth of *S. aureus* in a dose-dependent manner, with concentrations >0.005 % w/v being inhibitory, and concentrations between 0.025–0.000039 % w/c/ affecting the growth rate (Fig. S9). The growth of *P. aeruginosa* and *E. coli* was inhibited by 2 % w/v HEPB. All other concentrations tested affected the final OD in a dose-dependent manner (Fig. S4 and S7), although the growth rate of *E. coli* did not seem affected with concentrations <0.5 % w/v, while the growth rate of *P. aeruginosa* was affected with all the concentrations tested (Fig. S4). The effect of HEPB on the growth of *S. aureus* was different with concentrations of 2 to 0.5 % w/v affecting bacterial growth but not inhibiting it, while concentrations <0.25 % w/v had no effect on bacterial growth (Fig. S10).

### Change in susceptibility profile following exposure to preservatives

Baseline (i.e. pre-exposure) data for the three bacteria against the four preservatives were determined using a microdilution protocol based on ISO 20776–1 and are shown in Table 1. No MIC or MBC were obtained for TRI and *P. aeruginosa* since this bacterium is intrinsically resistant to TRI. *P. aeruginosa *, *S. aureus* and *E. coli* were not susceptible to HEPB at the highest concentration of 2 % w/v tested. Changes in the susceptibility profile of the bacteria were observed following 24 h exposure to BZC and TRI. Fold changes in MIC or MBC were calculated to help compare MIC or MBC values between pre-exposure and post-exposure (Table 1). A 24 h bacterial exposure to BZC increased MIC between 1.5- and 8-fold overall. For MBC values an increase of 2- to 2.7-fold was recorded for *P. aeruginosa* and *E. coli *, but the MBC marginally decreased for *S. aureus* by 0.4-fold (Table 1). TRI had a more adverse effect on significantly increasing *S. aureus* and *E. coli* MIC and MBC by >21- and 52-fold, respectively. Changes in bacterial susceptibility profile for HEPB at concentrations above 2 % w/v could not be tested.

**Table 1.** Mean MIC and MBC for the three test bacteria before and after 24 h exposure to BZC, TRI or HB at 20 °C (±1 °C). Exposure concentrations for each biocide/micro-organism combination are given in the text. Fold change was calculated by dividing MIC or MBC values after exposure with the values obtained pre-exposure. nd: not determined

	Pre-exposure		Exposure 24 h		Fold change in	
	**MIC %**	**MBC%**	**MIC%**	**MBC%**	**MIC**	**MBC**
**BZC**						
* S. aureus *	0.00078	0.0036	0.0063	0.0017	8	0.4
* E. coli *	0.0021	0.0031	0.0031	0.0083	1.5	2.7
* P. aeruginosa *	0.0063	0.013	0.025	0.025	4	2
**Triclosan**						
* S. aureus *	0.00042	0.00021	≥0.005	≥0.005	>12	>21
* E. coli *	0.00023	0.00025	0.0013	0.0013	5.7	52
* P. aeruginosa *	≥0.005	≥0.005	≥0.005	≥0.005	nd	nd
**Hydroxyethoxy phenyl butanone**						
* S. aureus *	≥2	≥2	≥2	≥2	nd	nd
* E. coli *	≥2	≥2	≥2	≥2	nd	nd
* P. aeruginosa *	≥2	≥2	≥2	≥2	nd	nd

Changes in the bacterial antibiotic susceptibility profiles following 24 h exposure to preservatives are shown in Table 2. Exposure of *E. coli* to TRI (0.00002%) altered the antibiotic susceptibility profile to tobramycin sufficiently to change the clinical interpretation from susceptible to intermediate. *S. aureus* exposure to preservatives for 24 h did not affect its antibiotic susceptibility profile. For all three preservatives, exposure for 24 h altered the antibiotic susceptibility profile of gentamicin sufficiently for change in clinical interpretation from intermediate to susceptible. *P. aeruginosa* became susceptible to ciprofloxacin following exposure to BZC or TRI (Table 2).

**Table 2.** Changes in antibiotic susceptibility profile following 24 h exposure to BZC, TRI or HB at 20 °C (±1 °C). Exposure concentrations for each biocide/bacteria combination are given in Table 1. Where the antibiotic is named, a change to that antibiotic susceptibility profile was measured (S: sensitive and I: intermediate) according to BSAC breakpoints [19]

Bacteria	Change in antibiotic susceptibility profile after 24 h exposure to		
	BZC	TRI	HEPB
* E. coli *	GEN (S)	GEN (S); TOB (I)	GEN (S)
* P. aeruginosa *	CIP (S)	CIP (S)	–
* S. aureus *	–	–	–

–, no change in susceptibility; AMP, ampicillin; BZC, Benzalkonium chloride; CIP, ciprofloxacin; GEN, gentamicin; HEPB, HydroxyEthoxy Phenyl Butanone; (I), becoming clinically intermediate; (S), becoming clinically susceptible; TIM, ticarcillin/clavulanic acid; TOB, tobramycin; TRI, triclosan.

## Discussion

This study aimed at measuring the effect of 24 h exposure to biocide on the change in antimicrobial susceptibility in three test bacteria. To date, there is no standard protocol to measure the impact of biocide and bacterial resistance and cross-resistance. Our protocol allows for exposing bacteria to a compound under ‘during use’ conditions [9], which reflect the worst-case scenario of product usage such as dilution or prolonged exposure during application. Here the test compound HEPB is intended to be used as a cosmetic preservative agent at a concentration currently permitted in EU (0.7 % w/v), hence the long exposure time of 24 h. Exposure concentration was chosen as 1/10 of the MIC as a worst-case scenario. The major limitation of the study was the inability to determine MIC and MBC values for HEPB, which were both >2 % w/v for the bacteria tested. This concentration was the highest concentration that could be tested at the time the experiments were conducted. Although, the alterations of MIC/MBC following 24 h exposure to HEPB (0.2 % w/v) could not be determined against the bacteria tested, the effects of this molecule in altering microbial growth and antibiotic susceptibility profile were determined. Exposure to HEPB at a 0.2 % w/v concentration for 24 h did not significantly affect the antibiotic susceptibility profile of the three bacteria. However, it did affect the growth kinetic of *P. aeruginosa* and *E. coli* at a concentration ranging 0.016–1 % and 0.13–1 % w/v, respectively. There were differences in the observed ‘inhibitory’ concentrations between the Bioscreen Growth Analyser and the ISO 20776–1 protocol, reflecting on the impact of using different protocols on measuring antimicrobial activity. The Bioscreen Growth Analyser is a useful automated system that provides additional information on the effect (other than inhibitory) of a compound on bacterial growth. It is particularly useful to detect extended lag phase and changes in growth kinetics during the log phase [17]. HEPB altered the growth kinetics of all bacteria in a concentration-dependent manner, indicating a detrimental effect on bacterial growth. No MIC was found using the standardized ISO method, although a 2 % w/v concentration inhibited the growth of both *P. aeruginosa* and *E. coli* using the Bioscreen Growth Analyser.

Different outcomes were observed with the exposure of the bacteria to BZC and TRI. Changes in antimicrobial susceptibility profile in Gram-negative bacteria following exposure to BZC have been reported with short contact time (5 min) and low concentrations (0.0001–0.005 % w/v) of the QAC 10, 15].

The increase in MIC or MBC following exposure to BZC was less than 10-fold in all the micro-organisms tested. There was no decrease in antibiotic susceptibility in all three bacteria. TRI produced more pronounced increase in MIC and MBC in the test bacteria, notably in *S. aureus* with a >12- and >21-fold increase, respectively (Table 3). Wesgate *et al*. [9] observed lower increase in MIC and MBC following 24 h exposure to TRI at an exposure concentration of 0.0004 % w/v. With *E. coli,* a 32-fold increase in MIC was observed [9] while in this study only 6-fold increase was observed although an increase in MBC was more significant with a 52-fold. Thus, it appears that using the same protocol, the extent in MIC and MBC increase depends on the exposure concentration. In addition, an increase in MIC/MBC was observed when the exposure concentration corresponded to a concentration that affected bacterial growth (Table 3). Wesgate *et al*. [9] already observed that MIC/MBC fold changes depended on contact time. The concept of a *minimum selective concentration* (MSC) for antibiotics [21] that spans a range of concentrations seems also to be the case for biocides. Our findings on the change in susceptibility profile to antibiotics when exposed to TRI for 24 h are broadly in concordance with those by Wesgate *et al*. [9], except for an increase in tobramycin MIC in *E. coli *. Change in bacterial susceptibility profile when exposed to TRI has been well documented [3, 4, 10, 12, 22] and justifies the use of the TRI as a positive control for this type of study. Muller *et al*. [23] observed that TRI (20 µM) was associated with an increased growth rate in *P. aeruginosa* in the presence of tetracycline, piperacillin and chloramphenicol, but TRI at 2 or 20 µM did not affect *P. aeruginosa* growth rate without antibiotics. This contrasts with our results with lower concentrations of TRI affecting *P. aeruginosa* growth rate. Unfortunately, we did not investigate the mechanisms responsible for the observed increase in MIC/MBC to the biocides or antibiotics. One reported global mechanism that can be associated with increased MIC/MBC to biocides and antibiotics in bacteria is the expression of efflux pumps (4, 22, 24–28).

**Table 3.** Exposure concentration used and effect of biocides on microbial growth

Microorganisms	Biocide	Range of concentration affecting growth (%w/v)*	24 h exposure concentration (%w/v)†	Increase in MIC or MBC‡	
				MIC	MBC
* P. aeruginosa *	BZC	0.0004–0.0063	0.00063	<4 folds	<4 folds
	TRI	0.01	0.001	–	–
	HB	0.004–1	0.2	–	–
* E. coli *	BZC	0.0016	0.00016	<4 folds	<4 folds
	TRI	0.00002–0.0025	0.00002	>4 folds	>20 folds
	HB	1	0.2	–	–
* S. aureus *	BZC	0.00004–0.0002	0.00008	>4 folds	no increase
	TRI	0.0004–0.0025	0.00005	>4 folds	>20 folds
	HB	0.5–2	0.2	–	–

*Range of concentration affecting growth determined with the bioscreen microbial growth analyser

†Exposure concentration defined as 1/10 of MIC determined with the ISO: 20776–1 (2006) microdilution protocol

‡–: not determined

Finally, our study investigated the inherent activity of biocides, but not that of formulated biocides. Formulations can negate or decrease the risk of developing bacterial resistance following treatment or repeated exposure [9, 10, 29].

Here, a new protocol was designed and used to provide information on the ability of a biocide or biocidal product to be associated with emerging antimicrobial resistance [2, 9, 10]. To this end, the use of TRI as a positive control was particularly appropriate. One limitation of the test for products under development is perhaps the difficulty to predict their conditions of use. This protocol relies on the concept of ‘during use’ exposure that define both concentration and exposure time of the product during use. This enables to set up a worst-case scenario for product usage in terms of dilution of the product and extended contact time. It is clear from the literature, that sub-MIC concentration and long contact time are usually more prone to a stable change in antimicrobial phenotype [3, 9, 12, 30], although a short contact time of 5 min could also be associated with significant MIC/MBC increases and changes in antibiotic clinical susceptibility [9, 10]. Measuring the effect of biocide during bacterial growth with this protocol based on phenotypic changes offers additional information on the global effect of biocide on bacteria. Although MIC determination based on the ISO: 20776–1 (2006) microdilution protocol is not directly comparable to the inhibition of growth measured with the Bioscreen microbial growth analyser, the use of the latter protocol might be useful to define the test concentrations that may affect resistance phenotypes.

Using this method, HEPB, a new molecule developed by L’Oréal R and I, intended to be used in cosmetic products as a new preservative, did not show changes in the antimicrobial susceptibility profile in the tested bacteria.

## Supplementary Data

Supplementary material 1
