# Supplementary material for: Hydroxyethoxy phenyl butanone, a new cosmetic preservative, does not cause bacterial cross-resistance to antimicrobials
Source: J Med Microbiol. 2020 Mar 18;69(5):670–5. doi: 10.1099/jmm.0.001147 (PMC7451044; doi:10.1099/jmm.0.001147)

Supplementary material

Figure S$_1$. Growth kinetics of *S. aureus*, *E. coli* and *P. aeruginosa* in broth only: ■*S. aureus*, ■*P. aeruginosa* ■*E. coli*, and in broth with 5% v/v DMSO: ■*S. aureus*, ■*E. coli*, ■*P. aeruginosa*.

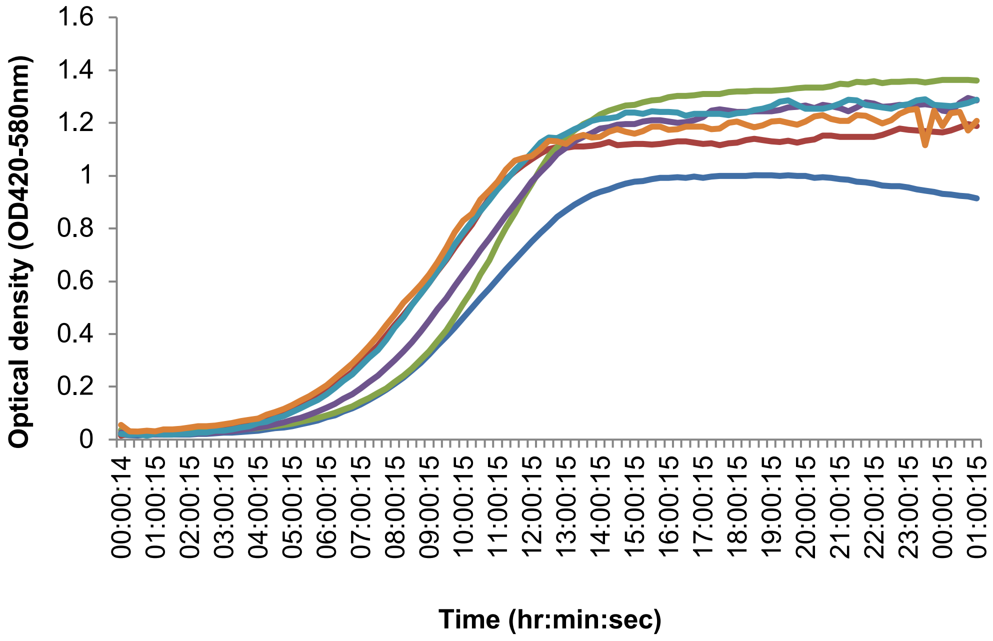

Figure S2 Growth kinetics of *Ps. aeruginosa* in benzalkonium chloride (n=15). Concentrations (% w/v): 0.05, 0.025, 0.013, 0.0063, 0.0031, 0.0016, 0.00078, 0.00039, 0.00019, 0.000098.

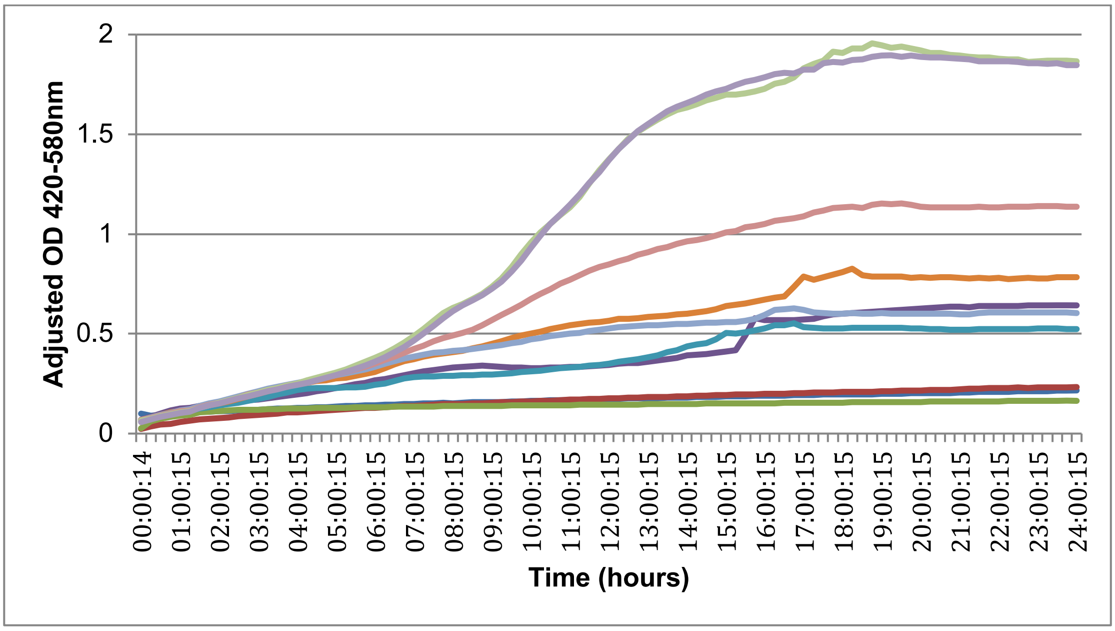

Figure S3 Growth kinetics of *Ps. aeruginosa* in triclosan in DMSO (n=15). Concentrations (% w/v): 0.01, 0.005, 0.0025, 0.0013, 0.00063, 0.00031, 0.00016, 0.000078, 0.000039, 0.000019, 5%DMSO.

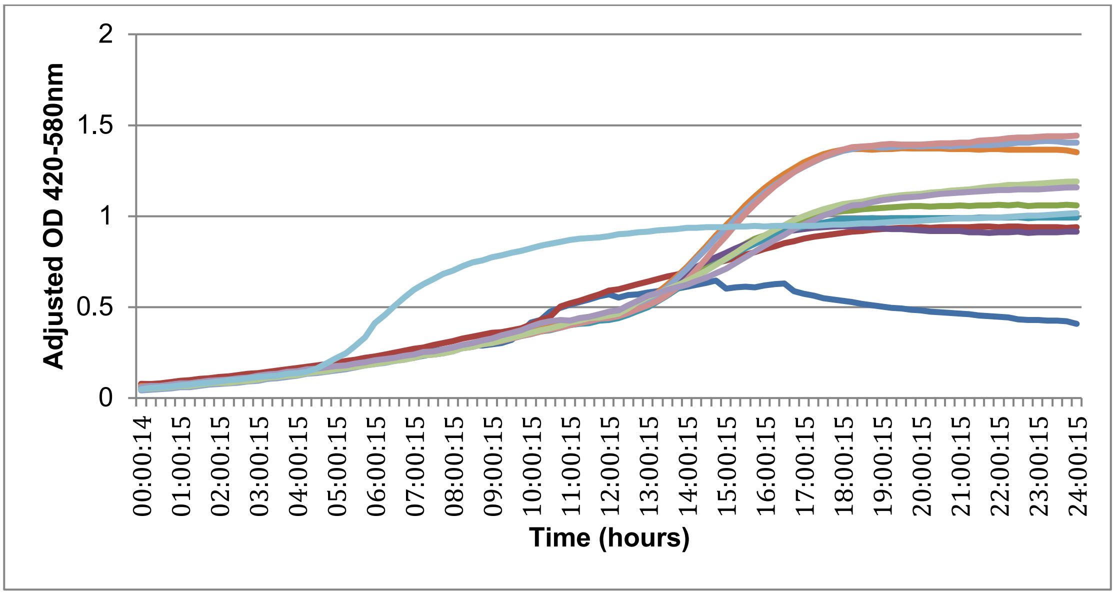

Figure S4 Growth kinetics of *Ps. aeruginosa* in hydroxyethoxyphenyl butanone in DMSO (n=15). Concentrations (% w/v): ■2, ■1, ■0.5, ■0.25, ■0.13, ■0.063, ■0.031, ■0.016, ■0.0078, ■0.0039, ■5%DMSO.

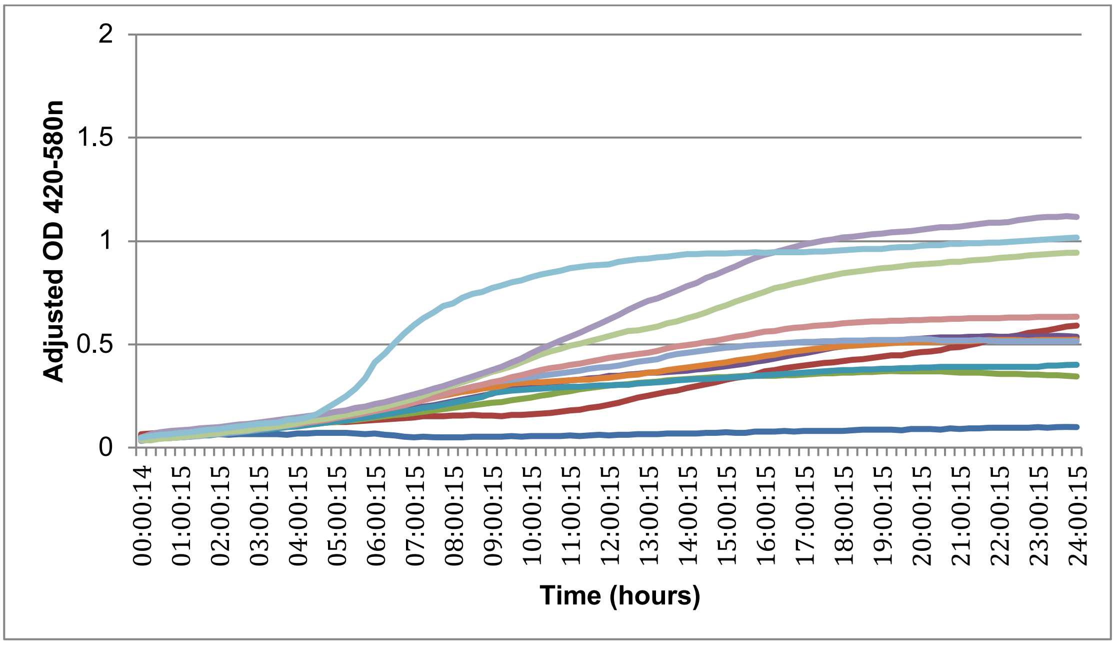

Figure S5 Growth kinetics of *E. coli* in benzalkonium chloride (n=15). Concentrations (% w/v): ■0.05, ■0.025, ■0.013, ■0.0063, ■0.0031, ■0.0016, ■0.00078, ■0.00039, ■0.00019, ■0.000098.

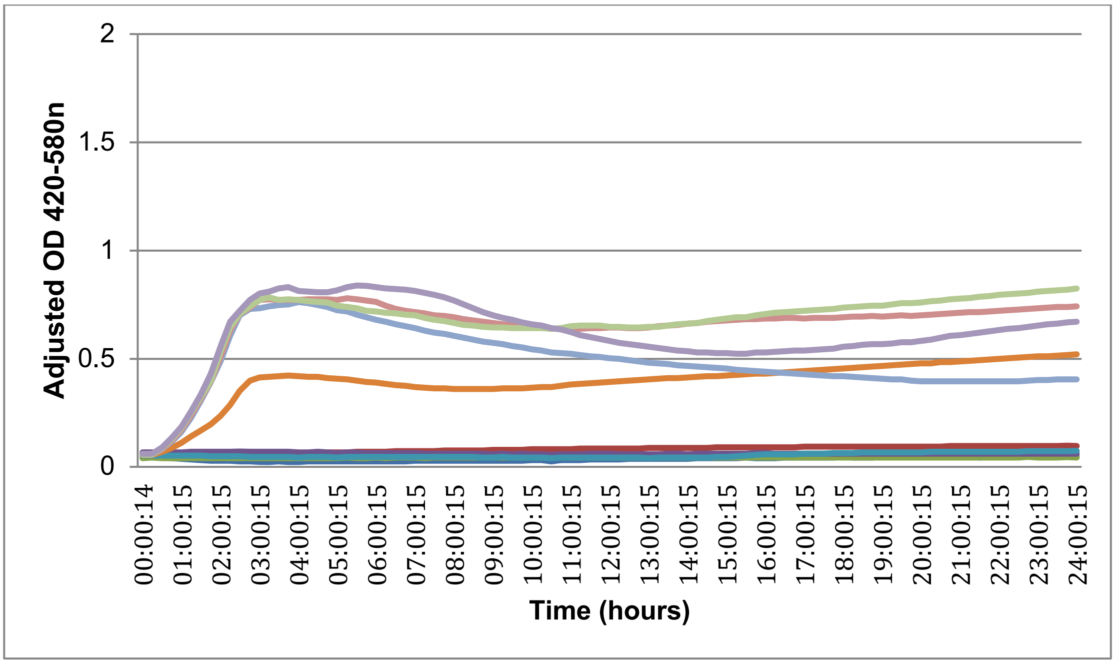

Figure S6 Growth kinetics of *E. coli* in triclosan in DMSO (n=15). Concentrations (% w/v): ■0.01, ■0.005, ■0.0025, ■0.0013, ■0.00063, ■0.00031, ■0.00016, ■0.000078, ■0.000039, ■0.000019, ■5%DMSO.

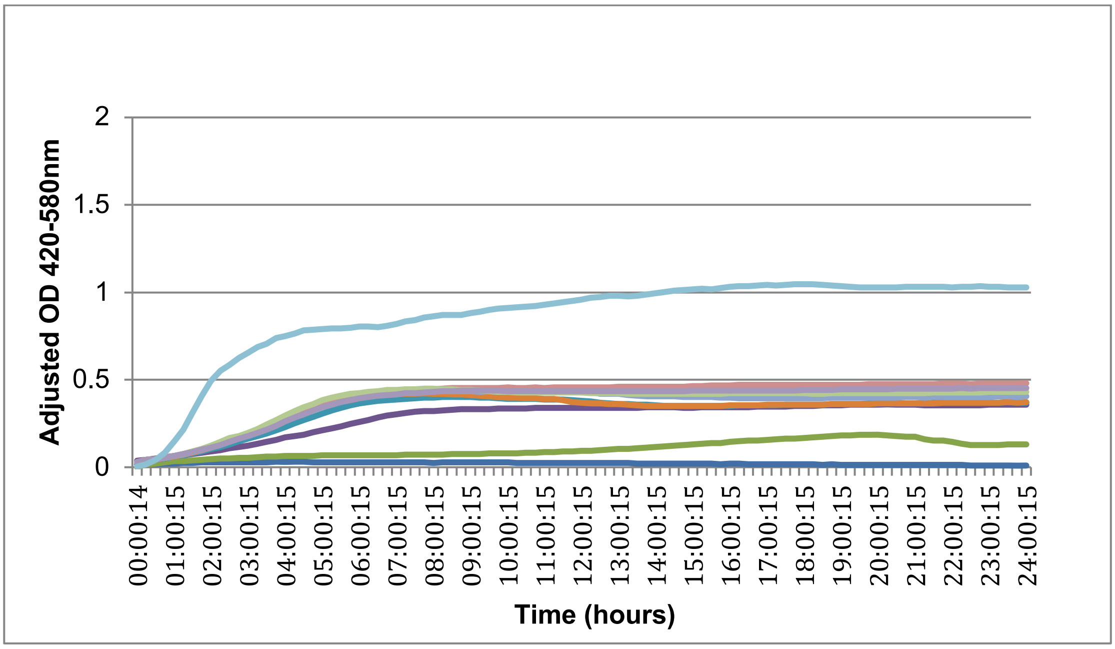

Figure S7 Growth kinetics of *E. coli* in hydroxyethoxyphenyl butanone in DMSO (n=15). Concentrations (% w/v): ■2, ■1, ■0.5, ■0.25, ■0.13, ■0.063, ■0.031, ■0.016, ■0.0078, ■0.0039, ■5%DMSO.

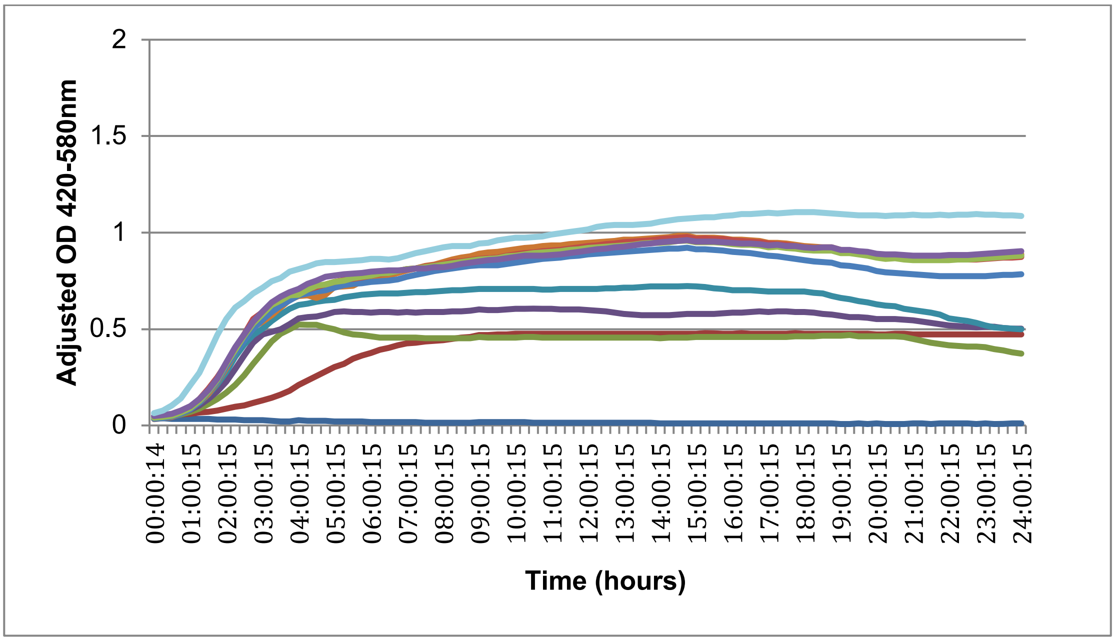

Figure S8 Growth Kinetics of *S. aureus* in benzalkonium chloride (n=15). Concentrations (% w/v): 0.05, 0.025, 0.013, 0.0063, 0.0031, 0.0016, 0.00078, 0.00039, 0.00019, 0.000098.

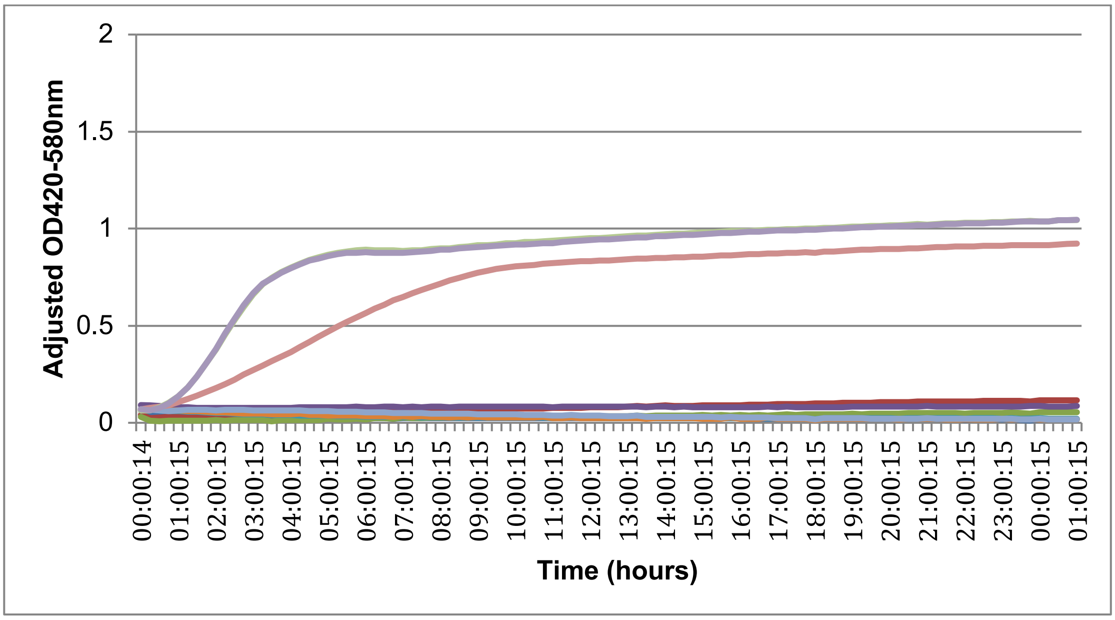

Figure S9 Growth kinetics of *S. aureus* in triclosan in DMSO (n=15). Concentrations (% w/v): 0.01, 0.005, 0.0025, 0.0013, 0.00063, 0.00031, 0.00016, 0.000078, 0.000039, 0.000019, 5%DMSO.

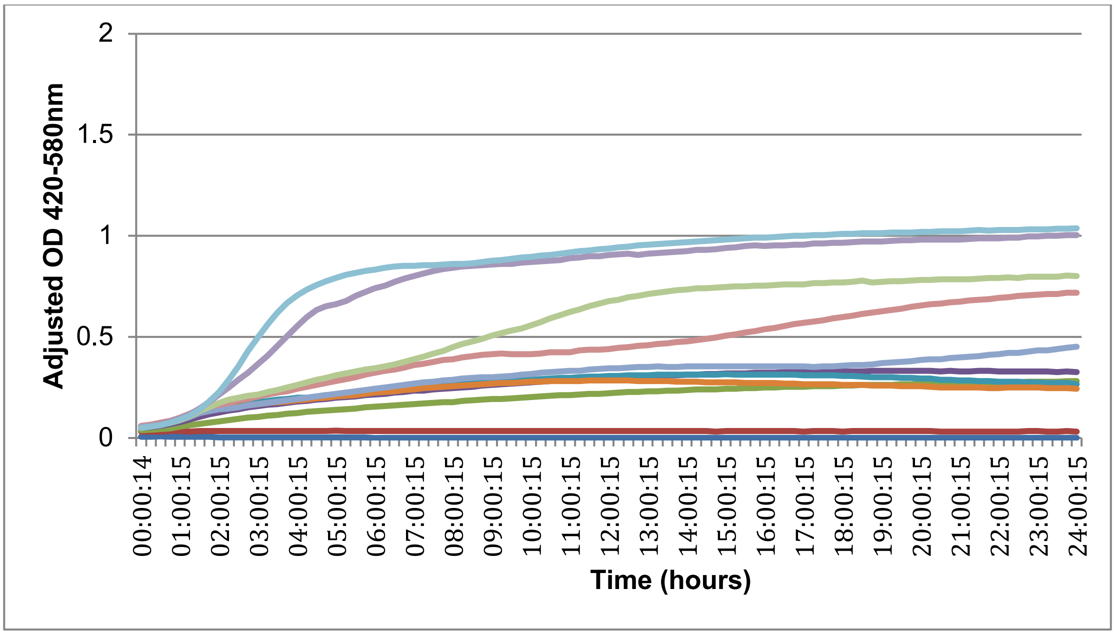

Figure S10 Growth kinetics of *S. aureus* in hydroxyethoxyphenyl butanone in DMSO (n=15) Concentrations (% w/v): ■2, ■1, ■0.5, ■0.25, ■0.13, ■0.063, ■0.031, ■0.016, ■0.0078, ■0.0039, ■5%DMSO.

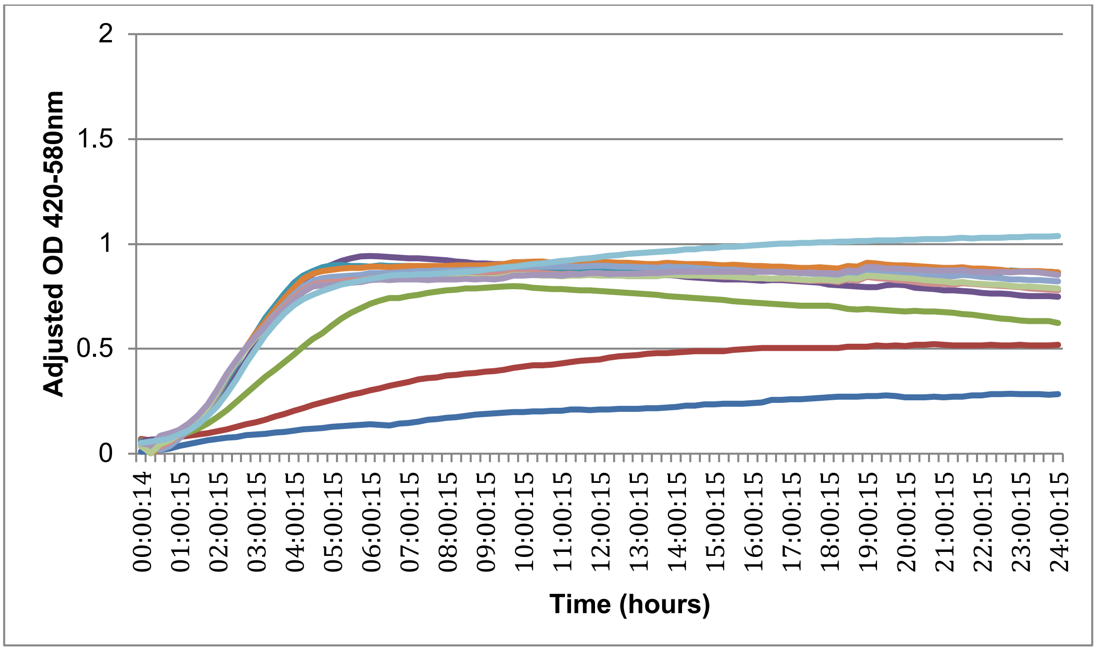

Supplement: Supplementary material 1 [file jmm-69-670-s001.pdf]
